# On-water surface synthesis of electronically coupled 2D polyimide-MoS_2_ van der Waals heterostructure

**DOI:** 10.1038/s42004-023-01081-3

**Published:** 2023-12-16

**Authors:** Anupam Prasoon, Hyejung Yang, Mike Hambsch, Nguyen Ngan Nguyen, Sein Chung, Alina Müller, Zhiyong Wang, Tianshu Lan, Philippe Fontaine, Thomas D. Kühne, Kilwon Cho, Ali Shaygan Nia, Stefan C. B. Mannsfeld, Renhao Dong, Xinliang Feng

**Affiliations:** 1 Center for Advancing Electronics Dresden (cfaed) and Faculty of Chemistry and Food Chemistry, Technische Universität Dresden, 01062 Dresden, Germany; 2 Max Planck Institute of Microstructure Physics, Weinberg 2, Halle, D-06120 Germany; 3 Center for Advancing Electronics Dresden (CFAED) and Faculty of Electrical and Computer Engineering, Technische Universität Dresden, 01062 Dresden, Germany; 4 Department of Chemical Engineering, Pohang University of Science and Technology, Pohang, 37673 Republic of Korea; 5 Synchrotron SOLEIL, L’Orme des Merisiers, Départementale 128, 91190 Saint-Aubin, France; 6 Center for Advanced Systems Understanding, Helmholtz-Zentrum Dresden-Rossendorf, 02826 Görlitz, Germany; 7 Institute of Artificial Intelligence, Chair of Computational System Sciences, Technische Universität Dresden, 01187 Dresden, Germany; 8 Key Laboratory of Colloid and Interface Chemistry of the Ministry of Education, School of Chemistry and Chemical Engineering, Shandong University, 27 Shandanan Road, Jinan, 250100 China

**Keywords:** Electronic materials, Surface assembly

## Abstract

The water surface provides a highly effective platform for the synthesis of two-dimensional polymers (2DP). In this study, we present an efficient on-water surface synthesis of crystalline monolayer 2D polyimide (2DPI) through the imidization reaction between tetra (4-aminophenyl) porphyrin (M1) and perylenetracarboxylic dianhydride (M2), resulting in excellent stability and coverage over a large area (tens of cm^2^). We further fabricate innovative organic-inorganic hybrid van der Waals heterostructures (vdWHs) by combining with exfoliated few-layer molybdenum sulfide (MoS_2_). High-resolution transmission electron microscopy (HRTEM) reveals face-to-face stacking between MoS_2_ and 2DPI within the vdWH. This stacking configuration facilitates remarkable charge transfer and noticeable n-type doping effects from monolayer 2DPI to MoS_2_, as corroborated by Raman spectroscopy, photoluminescence measurements, and field-effect transistor (FET) characterizations. Notably, the 2DPI-MoS_2_ vdWH exhibits an impressive electron mobility of 50 cm^2^/V·s, signifying a substantial improvement over pristine MoS_2_ (8 cm^2^/V·s). This study unveils the immense potential of integrating 2D polymers to enhance semiconductor device functionality through tailored vdWHs, thereby opening up exciting new avenues for exploring unique interfacial physical phenomena.

## Abstract (web summary)

The water surface has recently proven to be an effective platform for the synthesis of large-area two-dimensional polymers with high crystallinity. Here, the authors report the on-water synthesis of a crystalline monolayer 2D polyimide, as well as its incorporation into organic–inorganic hybrid van der Waals heterostructures that display significant charge transfer and high electron mobility.

## Introduction

Recent advancements in the realm of two-dimensional polymers (2DPs) have led to the emergence of a new generation of molecularly thin 2D materials that can be considered structural analogs of graphene^1–4^. These 2DPs exhibit extensive lateral dimensions and form covalent frameworks with long-range ordering in orthogonal directions^5,6^. Owing to their distinctive physicochemical properties, there has been a notable upsurge of interest in exploring potential applications for these materials, particularly in the realms of electronics, membranes, sensing, and various other domains^7–11^. Various synthetic approaches have been explored for the formation of single-layer 2DPs. The synthesis of 2DPs has primarily been achieved through exfoliation, chemical vapor deposition, and sol-gel techniques^5,7,12^. The formation of 2DPs on the water surface is particularly intriguing as it can directly provide monolayers^6,8,13,14^. In contrast to conventional bulk polymerization methods, 2D polymerization on the water surface is driven by the distinct conformations of monomers that interact with the surrounding environment, resulting in the self-assembly of extended, well-organized supramolecular structures^5,15,16^. Typically, amphiphilic monomers containing both hydrophilic and hydrophobic moieties are employed, and these monomers spontaneously arrange themselves on the water surface under the guidance of intermolecular forces such as hydrogen bonding, dipole-dipole interactions, and van der Waals forces. This intricate interplay of forces orchestrates the process of 2D polymerization, ultimately leading to the formation of highly crystalline 2D polymers^6,13,17^.

Thanks to their molecularly thin yet robust free-standing nature and their ability to be transferred over large areas (tens of cm^2^), 2DPs have opened exciting possibilities for designing van der Waals heterostructures based on 2DPs (2DP vdWHs), offering tunable band structures. These vdWHs are formed by assembling different atomically thin 2D materials, such as graphene, transition metal dichalcogenides (TMDs), and hexagonal boron nitride (h-BN), where the interactions between these layers are governed by weak van der Waals forces instead of strong covalent bonds^18–20^. These weak forces enable the precise tailoring of electronic and optoelectronic properties within vdWHs, leading to enhancements in carrier density, improved electron-hole separation, and accelerated charge transfer processes. The exceptional ability to control interlayer coupling in vdWHs has established a versatile platform for various applications, including innovative devices like tunneling transistors, photodetectors, and memory devices^21–31^. Although the focus of vdWHs has predominantly been on inorganic 2D materials, there is a relatively unexplored domain in this field concerning the integration of organic 2D crystals for the construction of organic-inorganic hybrid 2D vdWHs. Progress in developing such hybrid vdWHs has been hindered by the challenge of synthesizing well-defined monolayers of 2D polymers (2DPs) and precisely assembling them with other 2D materials in a predetermined sequence^6,32–35^. Thereby, the potential of 2DP-based vdWHs has remained largely untapped.

Herein, we present an efficient synthesis of a crystalline monolayer 2D polyimide (2DPI) through an imidization reaction between tetra (4-aminophenyl) porphyrin (M1) and perylenetracarboxylic dianhydride (M2) on the water surface under Langmuir-Blodgett (LB) conditions. In-situ synchrotron grazing incidence X-ray diffraction (GIXD) and surface-pressure area isotherm studies are used to elucidate the molecular orientation and self-assembly of the 2D polymerization on the water surface. Subsequently, the obtained high-quality, free-standing 2DPI film, covering a large cm^2^-scale area, was employed to fabricate an electronically coupled 2DPI-MoS_2_ vdWH with electrochemically exfoliated few-layer molybdenum sulfide (MoS_2_) through a wet transfer approach. GIXD and high-resolution transmission electron microscopy (HRTEM) manifested the face-to-face stacking arrangement between MoS_2_ and 2DPI in vdWH. Raman- and photoluminescence-spectroscopy studies, combined with electrical measurements in field effect transistors (FETs), revealed the significant charge transfer and n-type doping from monolayer 2DPI to MoS_2_ upon their contact. Additionally, these results demonstrate strong interlayer coupling, leading to enhanced device performance of the 2DPI-MoS_2_ vdWH, reaching a very high electron mobility of 50 cm^2^/V·s, superior to the pristine MoS_2_ with 8 cm^2^/V·s.

## Results and discussion

**Fig. 1:**
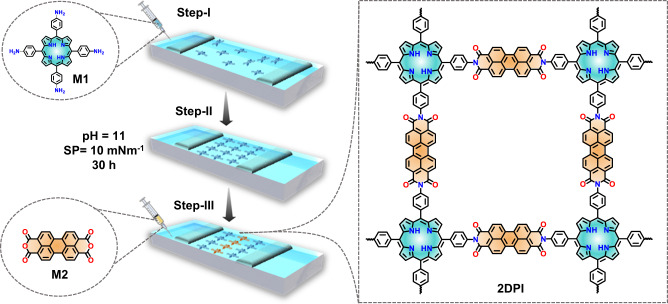
On-water surface 2D polymerization towards 2DPI monolayer. Step-by-step illustration of 2D polyimide 2DPI monolayer formation on the water surface via Langmuir-Blodgett conditions.

2D polyimide (2DPI) monolayers were synthesized on the water surface under Langmuir-Blodgett (LB) conditions, through the imidization reaction between M1 and M2 at room temperature, as depicted in Fig. 1. The synthesis process involved three steps: In Step-I, a chloroform solution of M1 (50 μL, 1 mg/mL) was spread onto the water surface in the LB trough. Furthermore, in Step-II, the Delrin barriers were compressed to create a densely packed M1 sub-monolayer at a surface pressure of 10 mN/m. Subsequently, in Step-III, M2 (20 mL, 1 mg/mL dissolved in a 1 mg/mL LiOH aqueous solution) was added to the water subphase. It was allowed to diffuse to the interface, initiating the 2D polymerization through imide bond formation between M1 and M2. After 30 hours of interfacial polymerization, a large area of shiny brownish-orange color film was obtained on the water surface.

### In-situ structural insight into the step-by-step assembly on the water surface

**Fig. 2:**
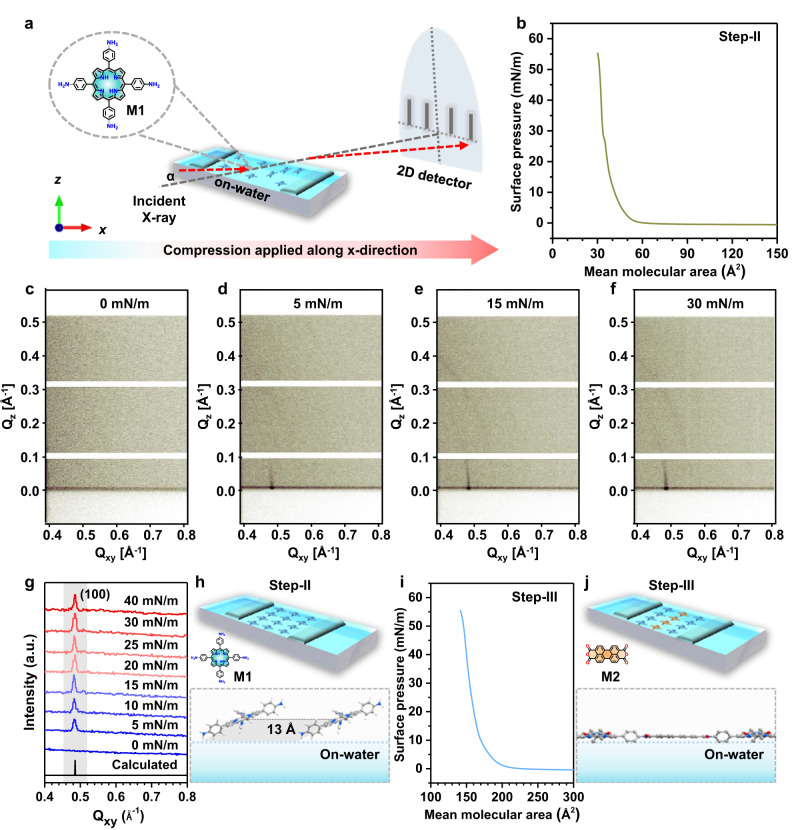
In-situ Grazing Incidence X-ray Diffraction (GIXD) on the water surface. **a** Illustration of the experimental setup for in-situ GIXD directly on the water surface. **b** Surface pressure area- isotherm of M1. **c**–**g** Surface-pressure dependent GIXD measurements of M1 on the water surface. **h** Schematic depicting the pre-assembled tilted orientation of M1 relative to the water surface. **i**, **j** Surface pressure-area isotherm after injecting M2 into the water sub-phase, showing the face-on orientation of 2DPI on the water surface.

To gain a deeper understanding of the on-water surface 2D polymerization process, we performed in-situ synchrotron surface pressure-dependent GIXD measurements directly on the water surface under Langmuir-Blodgett conditions, along with in-situ surface pressure vs. time measurements (Fig. 2a). The compression isotherm of M1 on the water surface revealed the formation of a stable monolayer with a relatively high collapse surface pressure of 55 mN/m and a mean molecular area (MMA) of 50 Å², suggesting the tilted orientation of M1 relative to the on-water surface (Fig. 2b). The structural evolution of M1 was measured with increasing surface pressure (π = 0, 5, 10, 15, 20, 25, 30, and 40 mN/m). The in-plane Bragg peak at Q_xy_ = 0.48 Å^-1^, d_100_ = 13 Å, corresponds to a 2D lattice structure with unit cell dimensions of a = b = 13 Å and γ = 90°. Taking into account that the size of M1 is approximately 15.7 Å, this implies that the monomers cannot lie flat on the water surface but are instead tilted relative to the water surface. Considering the π-π stacking distance of 4.0 Å, the calculated tilt angle is estimated to be about 18°. (Figs. 2c-2h, S1, S2). Notably, in the absence of any applied surface pressure (i.e., at 0 mN/m), the lack of a Bragg peak suggests the absence of a uniform molecular structure of M1 on the water surface (Fig. 2c). Therefore, certain surface pressure is needed to facilitate the assembly of M1 on the water surface. While increasing the surface pressure from 5 to 40 mN/m, the in-plane Bragg peak at Q_xy_ = 0.48 Å^-1^ was retained, demonstrating the robustness of the pre-assembled M1 structure on the water surface (Fig. 2g). Upon introducing M2 into the water sub-phase, the compression isotherm exhibited a significant increase in MMA, reaching 180 Å² (Fig. 2i). This notable shift in MMA is attributed to the face-on orientation of the 2DPI monolayer on the water surface, indicating the formation of imide bonds between M1 and M2, which in turn leads to the formation of 2D polymer. In addition, we closely monitored the evolution of the chemical reaction involving M1 and M2 using surface pressure measurements over the entire duration of the reaction (i.e., Step-II to Step-III). Once we achieved the self-assembled M1 monolayer with a notably high surface pressure, it remained stable even after 4 hours, indicating the high stability and robustness of the M1 monolayer on the water surface (Figures S3). Afterwards, when M2 was introduced into the water subphase, the surface pressure initially remained constant for approximately 7 hours, followed by a gradual increase and saturation after 18 hours, which corroborates the successful formation of 2DPI on the water surface (Figure S3).

**Fig. 3:**
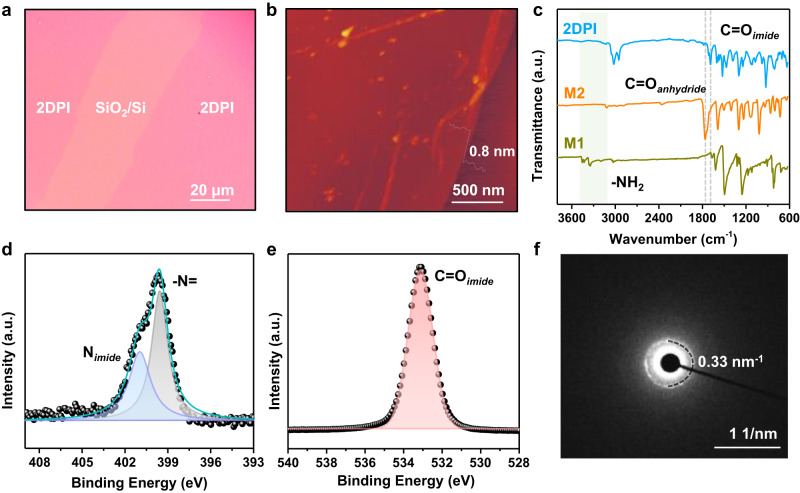
Characterizations of 2DPI. **a** An optical microscope image of the 2DPI film on a SiO_2_/Si substrate. **b** AFM image showing the monolayer features of the 2DPI. **c** ATR-FTIR spectroscopy of 2DPI shows the appearance of the imide C=O and the complete vanishing of the N-H stretch at ~3345 cm^-1^ from M1. **d**, **e** High-resolution XPS spectrum of the N1s region and the O1s region of 2DPI. **f** SAED pattern of the transferred monolayer 2DPI film.

The synthesized monolayer 2DPI film was transferred by the Langmuir-Schafer method to the substrate from the water surface for morphology and structural characterization. From the optical microscope image, 2DPI showed a uniform film (Fig. 3a). Atomic Force Microscopy (AFM) measurements revealed a thickness of approximately 0.8 nm, suggesting the formation of a monolayer 2DPI film (Fig. 3b). Attenuated total reflection Fourier-transform infrared spectroscopy (ATR-FTIR) spectra revealed significant changes: the peak corresponding to the N-H stretching of -NH_2_ (at 3345 cm^−1^) of M1 disappeared after polymerization. Simultaneously, the C = O vibration displayed a blue shift, transitioning from 1763 cm^−1^ (anhydride M2) to 1692 cm^−1^ (imide 2DPI)^6^ (Fig. 3c). In the Raman spectra of 2DPI, a distinctive peak attributed to the imide C−N bond appeared at 1403 cm^−1^ (Figure S4). Furthermore, the 2DPI film was analyzed using high-resolution X-ray photoelectron spectroscopy (XPS), and the high-resolution N1s peak at 401.1 eV confirmed the successful formation of the N imide, while at 399.5 eV, the characteristic –N= feature of M1 was observed (Fig. 3d). Additionally, the O1s peak at 533.1 eV demonstrated the successful formation of the C=O imide bond (Fig. 3e).

Collectively, the FTIR, XPS, UV-vis and Raman spectroscopy results clearly substantiate the targeted imide bond formation, along with the elimination of functional groups from the monomers. UV-vis absorption spectroscopy revealed that 2DPI exhibited the characteristic Soret band at 432 nm and Q bands at 523, 556, 599, and 655 nm (Figure S5). These findings support the successful synthesis of 2DPI. The crystalline structure of the 2DPI monolayer film was further elucidated using selected area electron diffraction (SAED) within a transmission electron microscope (TEM). The observation of a distinct diffraction spot at 0.33 nm^–1^ aligns perfectly with the (100) first-order reflections of 2DPI, providing evidence for the presence of a highly crystalline, long-range ordered structure in the synthesized monolayer film (Fig. 3f).

### Electronically coupled organic-inorganic hybrid van der Waals heterostructure

**Fig. 4:**
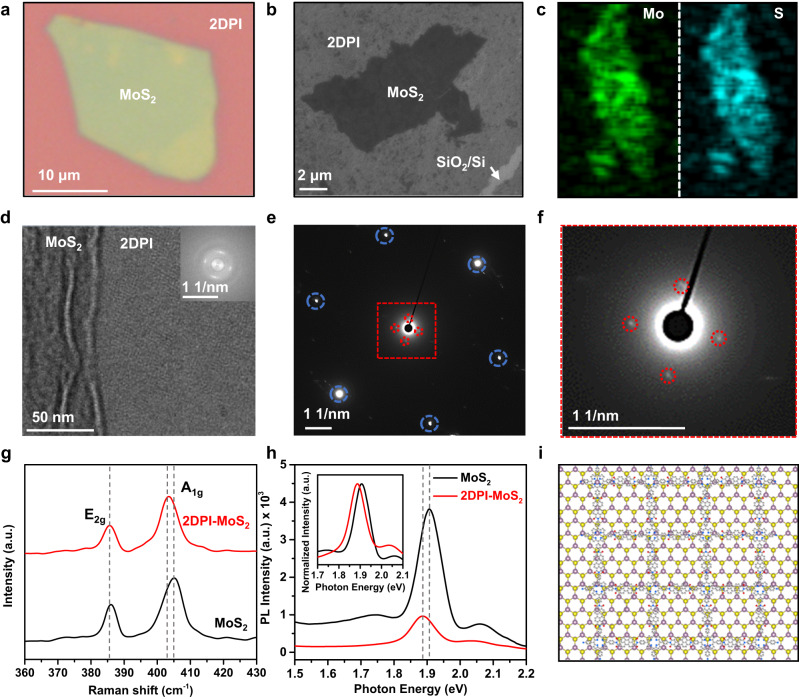
Electronically coupled 2DPI-MoS_2_ vdWH. **a** An optical microscope image of the 2DPI-MoS_2_ vdWH film on a SiO_2_/Si substrate. **b** FE-SEM images show uniform coverage of the 2DPI-MoS_2_ vdWH film. **c** Elemental mapping of Mo and S on the 2DPI-MoS_2_ vdWH film. **d**–**f** TEM and SAED images of the 2DPI-MoS_2_ vdWH film. **g**, **h** Raman spectra of 2DPI-MoS_2_ vdWH and the pristine MoS_2_ film. **h** PL spectra of 2DPI-MoS_2_ vdWH and the pristine MoS_2_ film. **i** Overlapping structure of 2DPI-MoS_2_ vdWH.

After successfully synthesizing high-quality molecularly thin free-standing films, 2DPI shows immense potential for creating vdWHs. These structures will allow the exploration of electronic properties in electronically coupled organic-inorganic hybrid vdWHs. The donor and acceptor moieties present in 2DPI offer a promising avenue for doping or functionalizing MoS_2_, leading to tailor-made engineered organic-inorganic hybrid vdWHs. The fabrication process of a 2DPI-MoS_2_ vdWH film involves combining a monolayer of 2DPI with exfoliated large-area MoS_2_ (~100 μm^2^) using a wet transfer method. The measured thickness of the exfoliated MoS_2_ flakes falls within the range of 7.9–8.6 nm (Figure S6). The resulting vdWH film was transferred onto the substrate and further annealed at 100 °C for 2 hours. The optical microscope image shows a vdWH film containing uniform 2DPI and MoS_2_ on a large scale (10 μm) (Fig. 4a). The surface morphology of the vdWH film, as shown in field-emission scanning electron microscopy (FE-SEM) images, exhibits uniform coverage of 2DPI-MoS_2_ vdWH film with contrast visible against the substrate (Fig. 4b). Elemental mapping reveals the presence of Mo and S elements across the vdWH film (Fig. 4c, S7). Additionally, the structure of 2DPI-MoS_2_ vdWH was characterized using TEM and SAED analysis. The TEM image reveals a distinct contrast between 2DPI and MoS_2_, and the corresponding fast Fourier transform (FFT) image clearly depicts the square lattice structure of 2DPI, featuring the (100) primary reflections of 2DPI (Fig. 4d). The SAED pattern from the overlapping 2DPI-MoS_2_ vdWH region exhibits two sets of diffraction spots, with the inner set corresponding to 2DPI and the outer set corresponding to MoS_2_ (Figs. 4e, f).

We employed Raman and photoluminescence spectroscopy to investigate the interfacial properties of the 2DPI-MoS_2_ vdWH, such as interlayer coupling and doping effects. The Raman spectra of pristine MoS_2_ displayed two distinct modes: the E_2g_ mode at 385.5 cm^–1^, associated with in-plane vibrations of Mo and S atoms, and the A_1g_ mode at 405.07 cm^–1^, related to out-of-plane vibrations of S atoms^36,37^. For the vdWH, a red shift of 1.22 cm^–1^ was observed in the A_1g_ mode of MoS_2_, while the E_2g_ mode remained unaltered (Fig. 4g). This red shift is attributed to the stronger coupling of the A_1g_ mode with electrons in the out-of-plane direction compared to the E_2g_ mode^37,38^. Additionally, this observation suggests a significant n-type doping influence in the MoS_2_ layer, primarily due to the electron-phonon coupling present in the 2DPI-MoS_2_ vdWH^36–40^. Figure 4h displays the photoluminescence (PL) spectra of pristine MoS_2_ and 2DPI-MoS_2_ vdWH. The PL spectra reveal a noticeable shift towards longer wavelengths, approximately 20 meV, and also a partial quenching of the intensity. This observed red shift and intensity decrease suggest the presence of charge transfer and n-doping effects^36–40^. These results are attributed to the unique band alignment at the interface of vdWH, which is consistent with the analysis obtained from Raman spectroscopy (Fig. 4i).

**Fig. 5:**
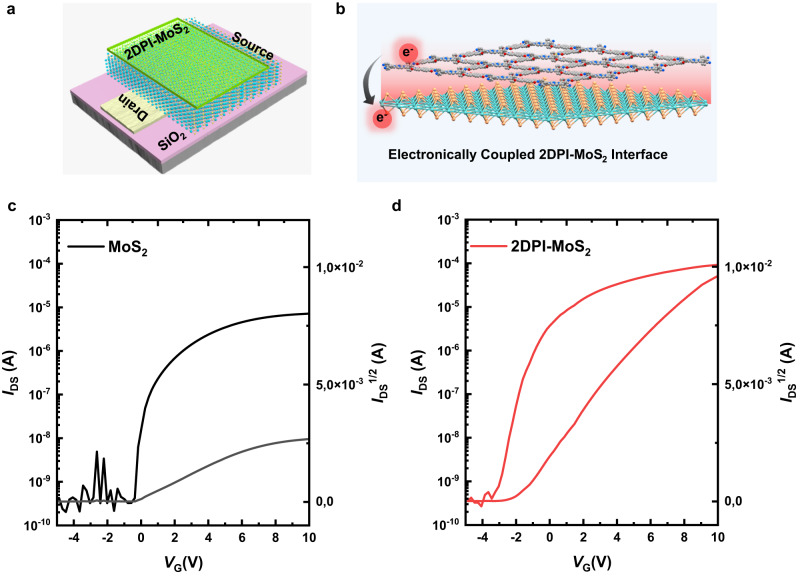
Electrical study of 2DPI-MoS_2_ vdWH. **a** Schematic illustration of FET devices employing 2DPI-MoS_2_ vdWH. **b** Schematic depiction of electronically coupled 2DPI-MoS_2_ interface. **c**, **d** FET transfer curve of pristine MoS_2_ and 2DPI-MoS_2_ vdWH devices.

To investigate the interfacial effects of charge transfer induced by a 2DPI, we further fabricated field-effect transistor (FET) devices based on pristine MoS_2_ and 2DPI-MoS_2_ vdWH (Figure S8). In this device configuration, source-drain charge transport occurred at the interface between the bottom dielectric SiO_2_ and the MoS_2_ layer (Fig. 5a). Both the FET devices of 2DPI-MoS_2_ vdWH and pristine MoS_2_ exhibited n-type characteristics with a high on/off ratio of approximately 10^5^ (Fig. 5c). However, the transfer curves of FET devices displayed significant differences. The pristine MoS_2_ FET exhibited an electron mobility of approximately 8 cm^2^/V^–s^, whereas the 2DPI-MoS_2_ vdWH FET displayed an electron mobility of 50 cm^2^/V^–s^ (Figs. 5c, 5d and for a detailed discussion, see Figure S9), which is well comparable to the state-of-the-art mobility achieved by the exfoliated MoS_2_ nanosheets. The considerably higher mobility observed in the 2DPI-MoS_2_ vdWH suggests more efficient charge transport in the MoS_2_ layer, attributed to the doping effect induced by the 2DPI layer on the MoS_2_ (Figure S10). The 2DPI contains an electron-rich donor unit, as porphyrin^41^, which can easily n-dope the MoS_2_, leading to enhanced electron current^35,42^. Additionally, the shift of the threshold voltage (V_th_) to a higher negative gate voltage (V_g_) in the 2DPI-MoS_2_ vdWH device compared to the MoS_2_ further supports the doping effect (Figs. 5c, d). This research opens up possibilities for utilizing 2D polymer-based heterostructures to enhance the functionality of semiconductor devices.

## Conclusion

In conclusion, we have successfully demonstrated the efficient synthesis of a highly crystalline 2DPI monolayer on the water surface using Langmuir-Blodgett conditions. Furthermore, we have fabricated an organic-inorganic 2DPI-MoS_2_ vdWH by integrating a monolayer of 2DPI with a few layers of MoS_2_. The resulting 2DPI-MoS_2_ vdWH exhibits strong interlayer coupling, significant charge transfer, and remarkably high electron mobility of 50 cm²/V^–s^, exceeding that of pristine MoS_2_ with 8 cm²/V^–s^. These findings highlight the substantial potential of these hybrid vdWH structures for advanced semiconductor devices. This research opens up possibilities for utilizing 2D polymer-based heterostructures in the realm of electronic and opto-electronic applications.

## Methods

The morphology and structure of the samples were investigated using optical microscopy (Zeiss), AFM (Bruker Multimode 8 HR), and HRTEM (JEOL Jem F-200C TEM with an acceleration voltage of 200 kV). For SEM measurements, thin films were deposited on a Si substrate, while copper grids were used for TEM measurements. Optical microscopy and AFM images were recorded on a 300-nm SiO_2_/Si substrate. UV-vis absorption spectra were acquired using a UV-vis-NIR spectrophotometer Cary 5000 device on a quartz glass substrate. Photoluminescence spectra were measured using the PerkinElmer fluorescence spectrometer LS 55. ATR-FTIR analysis was performed on a Tensor II system (Bruker) equipped with an attenuated total reflection unit. The samples for ATR-FTIR were prepared by depositing thin films onto a copper foil. Time-dependent surface-pressure measurements were carried out using the Langmuir-Blodgett trough (KSV NIMA, Finland). The trough was equipped with a platinum Wilhelmy plate, a Teflon dipper, and a pair of Delrin barriers.

### Materials

The chemicals, namely 4,4’,4”,4”‘-(porphyrin-5,10,15,20-tetrayl) tetraaniline (M1), anthra[2,1,9-def:6,5,10-d’e’f’]diisochromene-1,3,8,10-tetraone (M2), and sodium oleyl sulfate (SOS), were acquired from PorphyChem, abcr GmbH, and Sigma-Aldrich. They were utilized without further purification. Purified water was obtained using a Milli-Q purification system (Merck KGaA). The substrates, such as 300 nm SiO_2_/Si wafers, quartz glass, and copper grids, were obtained from Microchemicals and Plano GmbH.

### Synthesis of monolayer 2DPI

2D polyimide (2DPI) monolayers were synthesized on the water surface under Langmuir-Blodgett (LB) conditions, through the imidization reaction between M1 and M2 at room temperature, as depicted in Fig. 1. The synthesis process involved three steps: In Step-I, a chloroform solution of M1 (50 μL, 1 mg/mL) was spread onto the water surface in the LB trough. Furthermore, in Step-II, the Delrin barriers were compressed to create a densely packed M1 sub-monolayer at a surface pressure of 10 mN/m. Subsequently, in Step-III, M2 (20 mL, 1 mg/mL dissolved in a 1 mg/mL LiOH aqueous solution) was added to the water subphase. It was allowed to diffuse to the interface, initiating the 2D polymerization through imide bond formation between M1 and M2. After 30 hours of interfacial polymerization, a large area of shiny brownish-orange color film was obtained on the water surface.

### In-situ on-water surface GIXD

In-situ grazing-incidence X-ray diffraction (GIXD) measurements were performed at the SIRIUS beamline at SOLEIL, France. The beam energy was 8 keV and the size of the beam had dimensions of 2000 μm horizontally and 150 μm vertically. The incidence angle of the beam was 2 mrad. The measured films were grown on the air-water interface in a Langmuir trough with adjustable barriers, which was inside a helium-filled enclosure to reduce air scattering and beam damage to the film. The measurements were performed by scanning a Dectris Pilatus 1 M area detector with Soller slits (0.06° resolution) along the in-plane, horizontal angle (2θ) to record individual images (exposure times 5 s). The images were then horizontally integrated to obtain the vertical intensity distribution I(Q_z_) at each 2θ angle. These 1D spectra were then combined to create a 2D intensity map (Q_xy_-Q_z_) that was analyzed using WxDiff. The setup was calibrated using a film of behenic acid at the water surface.

### Supplementary information

Supplementary Information

## Data Availability

The data that support the plots within this paper and other findings of this study are available from the corresponding author upon reasonable request.

## References

[1] Colson JW, Dichtel WR (2013). Rationally synthesized two-dimensional polymers. Nat. Chem..

[2] Kissel P (2012). A two-dimensional polymer prepared by organic synthesis. Nat. Chem..

[3] Novoselov KS (2004). Electric field effect in atomically thin carbon films. Science.

[4] Cote AP (2005). Porous, crystalline, covalent organic frameworks. Science.

[5] Dong R, Zhang T, Feng X (2018). Interface-assisted synthesis of 2D materials: trend and challenges. Chem. Rev..

[6] Liu K (2021). A two-dimensional polyimide-graphene heterostructure with ultra-fast interlayer charge transfer. Angew. Chem. Int. Ed..

[7] Evans AM (2021). Two-dimensional polymers and polymerizations. Chem. Rev..

[8] Sahabudeen H (2016). Wafer-sized multifunctional polyimine-based two-dimensional conjugated polymers with high mechanical stiffness. Nat. Commu..

[9] Uribe-Romo FJ, Dichtel WR (2012). Polymers stripped down. Nat. Chem..

[10] Wang H (2019). Recent progress in covalent organic framework thin films: fabrications, applications and perspectives. Chem. Soc. Rev..

[11] Wang Z (2022). On-water surface synthesis of charged two-dimensional polymer single crystals via the irreversible Katritzky reaction. Nat. Synth..

[12] Feng X, Schlüter AD (2018). Towards macroscopic crystalline 2D polymers. Angew. Chem. Int. Ed..

[13] Müller V (2018). A two-dimensional polymer synthesized at the air/water interface. Angew. Chem. Int. Ed..

[14] Jin Y (2020). Confined growth of ordered organic frameworks at an interface. Chem. Soc. Rev..

[15] Dai W (2016). Synthesis of a two-dimensional covalent organic monolayer through dynamic imine chemistry at the air/water. Interface. Angew. Chem. Int. Ed..

[16] Sahabudeen H, Dong R, Feng X (2019). Interfacial synthesis of structurally defined organic two-dimensional materials: progress and perspectives. Chimia.

[17] Liu K (2019). On-water surface synthesis of crystalline, few-layer two-dimensional polymers assisted by surfactant monolayers. Nat. Chem..

[18] Hunt B (2013). Massive Dirac fermions and Hofstadter butterfly in a van der Waals heterostructure. Science.

[19] Novoselov KS, Mishchenko A, Carvalho A, Castro Neto AH (2016). 2D materials and van der Waals heterostructures. Science.

[20] Geim AK, Grigorieva IV (2013). Van der Waals heterostructures. Nature.

[21] Liu Y (2016). Van der Waals heterostructures and devices. Nat. Rev. Mater..

[22] Liang SJ, Cheng B, Cui X, Miao F (2020). Van der Waals heterostructures for high-performance device applications: challenges and opportunities. Adv. Mater..

[23] Xu H (2014). High responsivity and gate tunable graphene-MoS_2_ hybrid phototransistor. Small.

[24] Jariwala D (2013). Gate-tunable carbon nanotube–MoS_2_ heterojunction pn diode. Proc. Natl. Acad. Sci. USA..

[25] Bertolazzi S, Krasnozhon D, Kis A (2013). Nonvolatile memory cells based on MoS_2_/graphene heterostructures. ACS nano.

[26] Georgiou T (2013). Vertical field-effect transistor based on graphene–WS2 heterostructures for flexible and transparent electronics. Nat. Nanotechnol..

[27] Zheng Q (2017). Phonon-assisted ultrafast charge transfer at van der Waals heterostructure interface. Nano Lett..

[28] Fu S (2023). Reversible electrical control of interfacial charge flow across van der Waals interfaces. Nano Lett..

[29] Roy K (2013). Graphene–MoS_2_ hybrid structures for multifunctional photoresponsive memory devices. Nat. Nanotechnol..

[30] Flöry N (2020). Waveguide-integrated van der Waals heterostructure photodetector at telecom wavelengths with high speed and high responsivity. Nat. Nanotechnol..

[31] Sierra JF, Fabian J, Kawakami RK, Roche S, Valenzuela SO (2021). Van der Waals heterostructures for spintronics and opto-spintronics. Nat. Nanotechnol..

[32] Wang H (2020). Creation of a two-dimensional polymer and graphene heterostructure. Nanoscale.

[33] Zhong Y (2019). Wafer-scale synthesis of monolayer two-dimensional porphyrin polymers for hybrid superlattices. Science.

[34] Balch HB (2020). Electronically coupled 2D polymer/MoS_2_ heterostructures. J. Am. Chem. Soc..

[35] Wang, C. et al. Enhancing the carrier transport in monolayer MoS_2_ through interlayer coupling with 2D covalent organic frameworks. *Adv. Mater*., 2305882 (2023). 10.1002/adma.202305882 37690084

[36] Li H (2012). From bulk to monolayer MoS_2_: evolution of Raman scattering. Adv. Funct. Mater..

[37] Mawlong LPL, Bora A, Giri PK (2019). Coupled charge transfer dynamics and photoluminescence quenching in monolayer MoS_2_ decorated with WS_2_ quantum dots. Sci. Rep..

[38] Li Z (2015). Graphene quantum dots doping of MoS_2_ monolayers. Adv. Mater..

[39] Lin L (2013). Fabrication of luminescent monolayered tungsten dichalcogenides quantum dots with giant spin-valley coupling. ACS Nano.

[40] Dhakal KP (2014). Confocal absorption spectral imaging of MoS_2_: optical transitions depending on the atomic thickness of intrinsic and chemically doped MoS_2_. Nanoscale.

[41] Zhang C (2017). Highly fluorescent polyimide covalent organic nanosheets as sensing probes for the detection of 2, 4, 6-trinitrophenol. ACS Appl. Mater. Interfaces.

[42] Han KH (2019). Reduction of threshold voltage hysteresis of MoS_2_ transistors with 3-aminopropyltriethoxysilane passivation and its application for improved synaptic behavior. ACS Appl. Mater. Interfaces.

