# Supplementary material for: On-water surface synthesis of electronically coupled 2D polyimide-MoS2 van der Waals heterostructure
Source: Commun Chem. 2023 Dec 16;6:280. doi: 10.1038/s42004-023-01081-3 (PMC10725426; doi:10.1038/s42004-023-01081-3)
Supplement: Supplementary file 1 — Supplementary Information [file 42004_2023_1081_MOESM1_ESM.pdf]

On-water surface synthesis of electronically coupled 2D polyimide-MoS_2 van der Waals heterostructure

Anupam Prasoon[1,2], Hyejung Yang[1], Mike Hambsch[3], Nguyen Ngan Nguyen[1,2], Sein Chung[4], Alina Müller[1], Zhiyong Wang[1,2], Tianshu Lan[1,2], Philippe Fontaine[5], Thomas D. Kühne[6,7], Kilwon Cho[4], Ali Shaygan Nia[1], Stefan C. B. Mannsfeld[3], Renhao Dong[1,8], Xinliang Feng[1,2]*

[1]Center for Advancing Electronics Dresden (cfaed) and Faculty of Chemistry and Food Chemistry, Technische Universität Dresden, 01062 Dresden, Germany

[2]Max Planck Institute for Microstructure Physics, Halle (Saale) D-06120, Germany

[3]Center for Advancing Electronics Dresden (cfaed) and Faculty of Electrical and Computer Engineering, Technische Universität Dresden, 01062 Dresden, Germany

[4]Department of Chemical Engineering, Pohang University of Science and Technology, Pohang, 37673 Republic of Korea

[5]Synchrotron SOLEIL, L'Orme des Merisiers, Départementale 128, 91190, Saint-Aubin, France

[6]Center for Advanced Systems Understanding, Helmholtz-Zentrum Dresden-Rossendorf, 02826 Görlitz, Germany

[7]Institute of Artificial Intelligence, Chair of Computational System Sciences, Technische Universität Dresden, 01187 Dresden, Germany

[8]Key Laboratory of Colloid and Interface Chemistry of the Ministry of Education, School of Chemistry and Chemical Engineering, Shandong University, 27 Shandanan Road, Jinan 250100, China

*E-mail: xinliang.feng@tu-dresden.de

Table of Contents

Supplementary Figure 1..3

Supplementary Figure 2..4

Supplementary Figure 3..5

Supplementary Figure 4..6

Supplementary Figure 5..7

Supplementary Figure 6..8

Supplementary Figure 7..9

Supplementary Figure 8..10

Supplementary Figure 9..11

Supplementary Figure 10..12

References..13

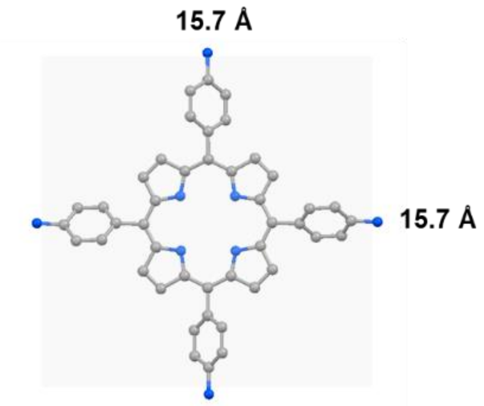

Supplementary Figure 1. The pre-organized M1 structure on the water surface exhibits the lattice parameters a = b = 13 Å and γ = 90°, which are significantly smaller than the size of an individual M1 molecule, a = b = 15.7 Å.

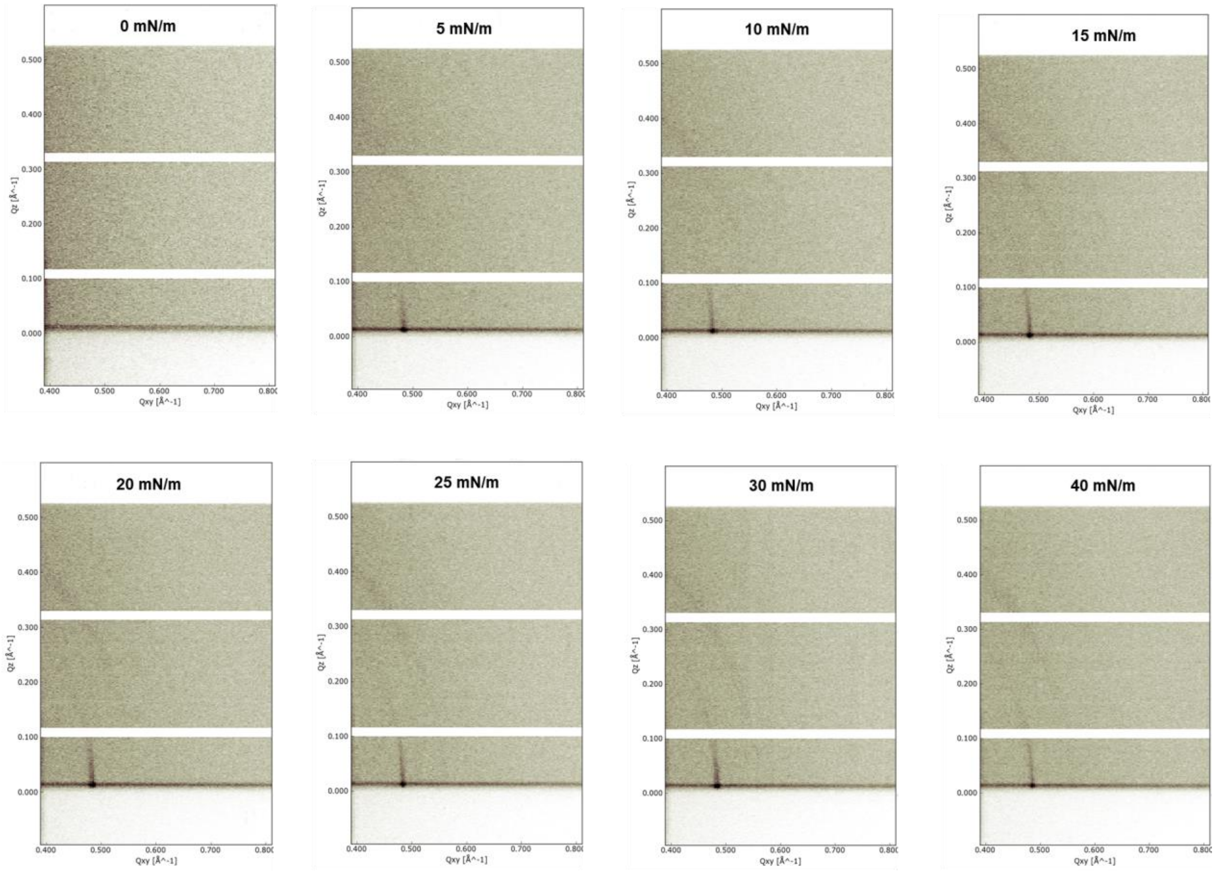

Supplementary Figure 2. *In-situ* surface-pressure dependent GIXD measurements of M1 on the water surface.

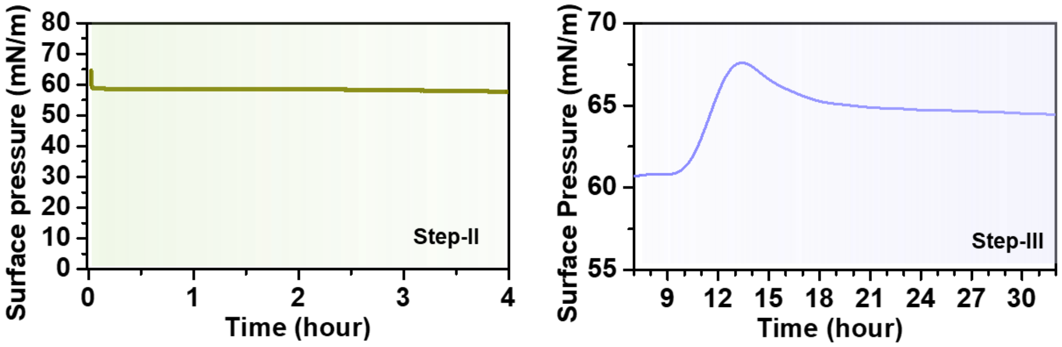

Supplementary Figure 3. The evolution of the chemical reaction involving M1 and M2 was monitored using surface pressure measurements versus time over the entire duration of the reaction, i.e., from Step-II to Step-III.

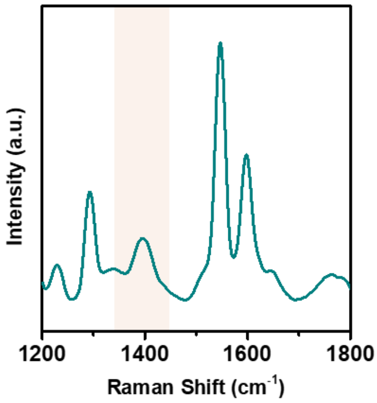

Supplementary Figure 4. The Raman spectra of 2DPI show a peak at ~1403 cm^{-1}, indicating the formation of an imide C-N bond.

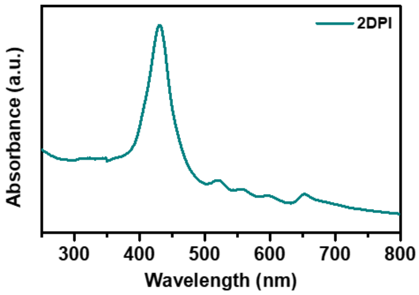

Supplementary Figure 5. Solid-state UV-Vis spectra of the as-synthesized 2DPI monolayer.

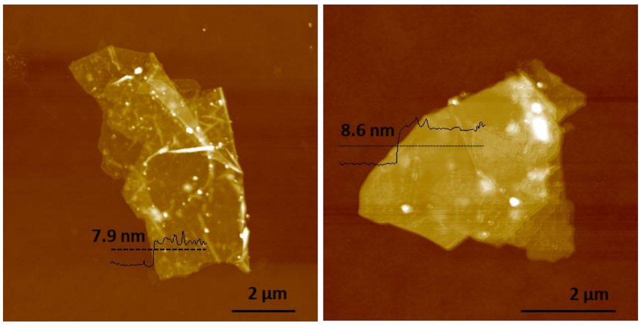

Supplementary Figure 6. AFM images of electrochemically exfoliated few-layer MoS_2.

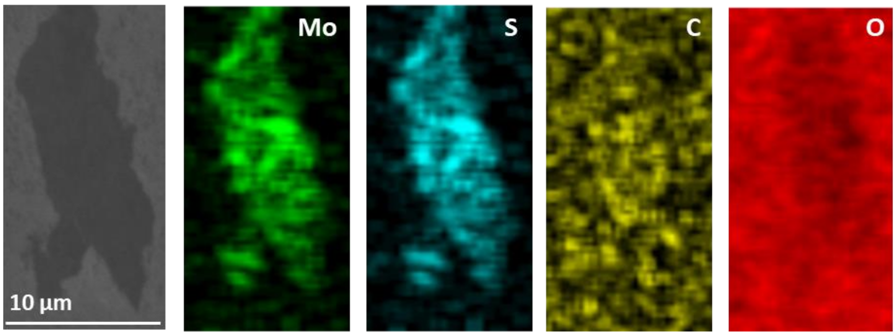

Supplementary Figure 7. Elemental mapping of Mo, S, C, and O was performed on the 2DPI-MoS_2 vdWH film.

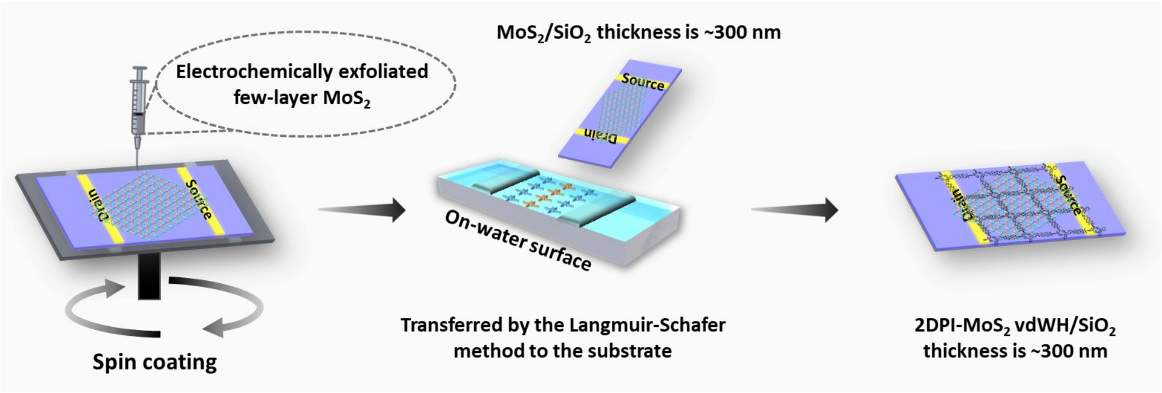

Supplementary Figure 8. Schematic of the preparation of 2DPI-MoS_2 vdWH by a wet transfer technique and the device configuration has a ¼ channel length/width ratio, and the SiO_2 thickness is approximately 300 nm.

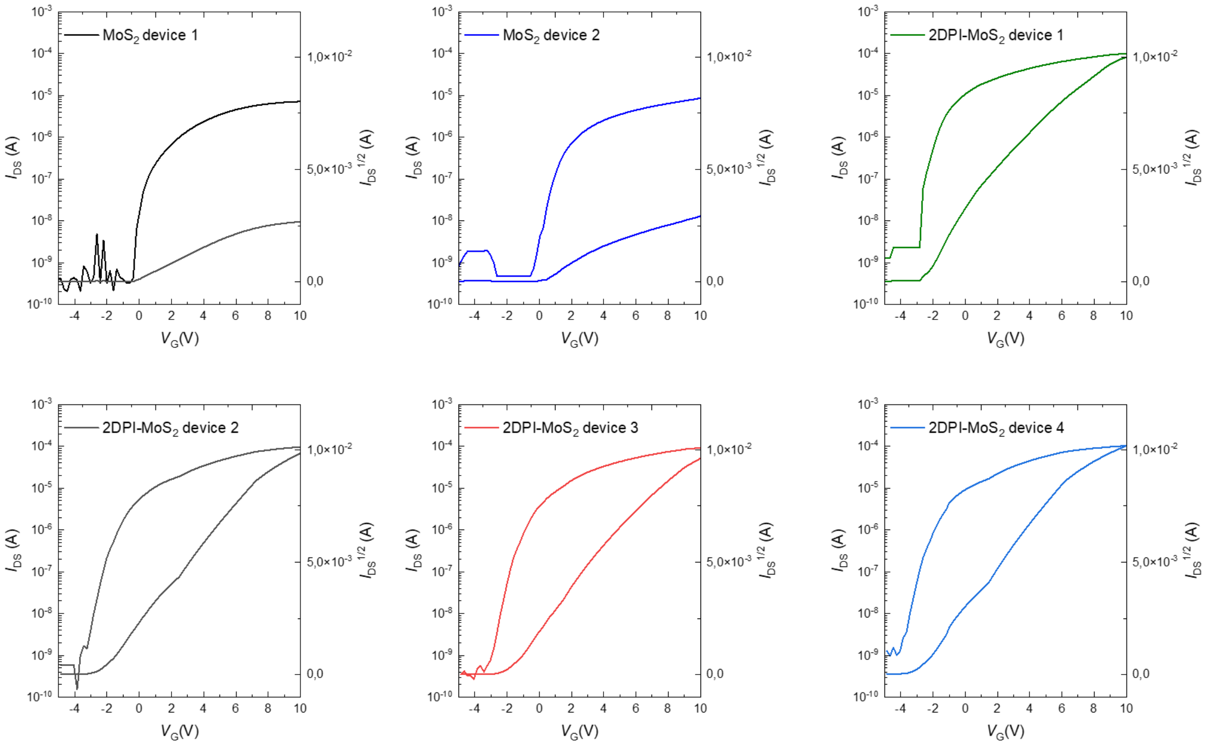

Supplementary Figure 9. FET transfer curve of pristine MoS_2 and 2DPI-MoS_2 vdWH devices.

	Mobility		Vth	
	MoS_2	2DPI-MoS_2	MoS2	2DPI-MoS_2
#1	5.4	50.0	0.0	-2.6
#2	8.0	34.4	0.5	-2.5
#3		39.6		-1.9
#4		42.3		-3.0
Average	6.72	41.59	0.23	-2.50

Supplementary Table T1. Table of statistical results for FET performance.

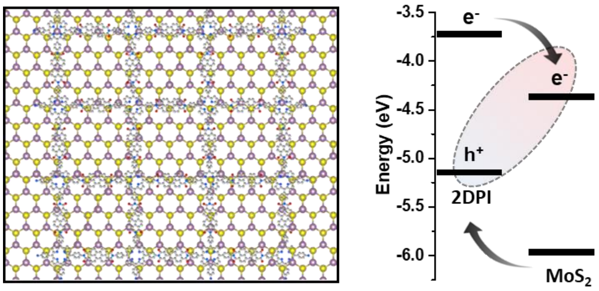

Supplementary Figure 10. Band alignment of 2DPI-MoS_2 vdWH[1,2].

The interactions between 2DPI and MoS_2

The high crystallinity of the monolayer 2DPI film offers a uniform periodic doping effect on MoS_2 in the 2DPI-MoS_2 vdWH. The Raman spectra of pristine MoS_2 displayed two distinct modes: the E_{2g} mode at 385.5 cm^{-1}, associated with in-plane vibrations of Mo and S atoms, and the A_{1g} mode at 405.07 cm^{-1}, related to the out-of-plane vibrations of S atoms. For the 2DPI-MoS_2 vdWH, a red shift of 1.22 cm^{-1} was observed in the A_{1g} mode of MoS_2, while the E_{2g} mode remained unaltered (Figure 4g). This red shift is attributed to the stronger coupling of the A_{1g} mode with electrons in the out-of-plane direction compared to the E_{2g} mode. The enhanced FET mobility of the 2DPI-MoS_2 vdWH can be attributed to the synergistic effect of the efficient interfacial electron transfer process and the pronounced suppression of the MoS_2 lattice vibration[3].

References

1. Dhakal, K. P. *et al.* Confocal absorption spectral imaging of MoS_2: optical transitions depending on the atomic thickness of intrinsic and chemically doped MoS_2. *Nanoscale* **6**, 13028-13035 (2014).

2. Zhang, C. *et al.* Highly fluorescent polyimide covalent organic nanosheets as sensing probes for the detection of 2, 4, 6-trinitrophenol. *ACS Appl. Mater. Interfaces* **9**, 13415-13421 (2017).

3. Wang, C. *et al.* Enhancing the carrier transport in monolayer MoS_2 through interlayer coupling with 2D covalent organic frameworks. *Adv. Mater.*, 2305882 (2023).
